# Chemical contaminants in donkey milk: A review of literature on sources, routes and pathways of contamination, regulatory framework, health risks, and preventive measures

**DOI:** 10.1016/j.heliyon.2024.e39999

**Published:** 2024-10-30

**Authors:** Dragana Ljubojević Pelić, Sava Lazić, Milica Živkov Baloš

**Affiliations:** Scientific Veterinary Institute “Novi Sad”, Rumenački put 20, 21000, Novi Sad, Serbia

**Keywords:** Food safety, Heavy metals, Aflatoxin M1, Pesticides, Polychlorinated biphenyls, Veterinary drugs

## Abstract

Donkey milk has garnered increasing attention due to its potential health benefits and nutritional properties, positioning it as a valuable alternative to cow's milk for specific consumer groups, such as individuals with allergies, young children, elderly populations, and those with compromised immune systems. However, the presence of chemical contaminants in donkey milk presents a significant concern for food safety and public health. This review aims to provide an assessment of the types and sources of chemical contaminants in donkey milk, including heavy metals, mycotoxins, pesticides, polychlorinated biphenyls, and antimicrobial and antiparasitic veterinary drugs. Through a comprehensive analysis of available literature, we examine the routes and pathways through which these contaminants enter the milk, their prevalence, and the associated health risks. The review also briefly discusses analytical methods for detecting these contaminants and the existing legislative framework that regulates these contaminants, underscoring its critical role in safeguarding public health and promoting safe consumption of donkey milk products. By identifying gaps in existing research and suggesting areas for further study, this review seeks to contribute to the development of more effective strategies for monitoring and mitigating chemical contamination in donkey milk, ultimately safeguarding consumer health and supporting the sustainable production of this niche dairy product.

## Highlights

• Donkey milk provides health benefits as an alternative to cow's milk.
• Chemical contaminants pose significant concerns for food safety and health.
• This is among the first reviews on chemical contaminants in donkey milk.
• The review emphasizes regulations and challenges in monitoring contaminants.
• Limited studies on contaminants in donkey milk highlight a need for further research.

## 1 Introduction

Donkeys (*Equus asinus*) are part of the family *Equidae*, which also includes horses, and zebras [1]. The species originates from the African wild ass and has been domesticated for thousands of years [2]. Donkeys are known for their resilience and adaptability to various environments, making them valuable livestock in many regions globally. There are several breeds of donkeys, varying in size, colour, and region of origin, which can influence their milk production and quality [3,4]. Donkey milk, traditionally used in some regions, has recently gained increased attention due to its nutritional and hypoallergenic properties [[4], [5], [6]]. It is particularly esteemed for its similarities to human breast milk [7], making it a suitable alternative for infants and individuals with cow milk allergies [[7], [8], [9], [10], [11], [12], [13]], which contribute to its health-promoting properties [[14], [15], [16], [17]]. In addition to its fresh form, donkey milk is available in condensed and powdered versions, which offer extended shelf life and ease of transport, making it a viable alternative in regions with limited access to fresh supplies [18,19]. These processed forms are created through dehydration techniques that preserve essential nutrients while reducing the moisture [20] and volume, thus facilitating broader distribution. However, concerns regarding potential chemical contaminants, such as heavy metals, pesticides, and other pollutants, remain significant, as these can remain during the manufacturing process, posing potential health risks to consumers. Polidori et al. [21] reported that the nutritional characteristics of fresh and freeze-dried donkey milk were essentially the same, yielding extremely similar results in terms of the two types of milks' chemical makeup, fatty acid profiles, and mineral contents. Their findings supported the idea that freeze-drying is a relatively sparing method of preserving perishable goods because it significantly lowers the transportation and storage expenses of freeze-dried milk while having little effect on the primary fresh milk quality parameters. Thermal treatments during milk processing are ineffective at breaking down the residues of heavy metals, pesticides or veterinary drugs, meaning that raw milk, heat-treated milk, and other dairy products can have comparable levels of residue contamination [22,23]. Despite its nutritional advantages, the safety and quality of donkey milk have requested the careful and detailed examination due to the potential presence of various contaminants. These contaminants, including heavy metals, mycotoxins, pesticides, polychlorinated biphenyls (PCBs) and residue of veterinary drugs can enter the milk through various routes, such as environmental pollution, agricultural practices, and metabolic pathways (e.g., AFM1 as a by-product of AFB1 in contaminated feed). In addition to contamination through feed and environmental exposure, inadequate storage and handling practices can further contribute to the levels of these contaminants in milk [[24], [25], [26], [27]]. The presence of such contaminants not only poses significant health risks to consumers but also undermines the therapeutic potential of donkey milk. This review will explore the sources and pathways of chemical contaminants in the animal body and during the processing stages, the levels of different contaminants commonly found in donkey milk, and the potential health risks associated with their consumption. Furthermore, the review will discuss existing regulatory frameworks for donkey milk, as well as different measures and strategies to minimize contamination. Through a detailed literature examination, the present review aims to highlight the critical need for ongoing monitoring and research to ensure donkey milk quality and safety in the context of chemical contamination. To date, limited research has been dedicated to assessing chemical contaminants specifically in donkey milk. This review is one of the first comprehensive efforts to address this gap, providing a detailed evaluation of various contaminants, including heavy metals, mycotoxins, pesticides, and veterinary drug residues. The novelty of this review lies in its focus on a niche but increasingly popular dairy product, highlighting the importance of establishing safety profiles and guiding future research. This review aims to provide a comprehensive overview of the current state of knowledge regarding chemical contaminants in donkey milk, their implications for food safety, and the methodologies employed for their detection and quantification.

## 2 Materials and methods

A comprehensive literature review was carried out using data from various sources. The databases utilized included Scopus, MDPI, Taylor and Francis, PubMed Wiley, Springer, and Web of Science. About 100 manuscripts were reviewed for this study. The manuscripts, published between 1995 and 2024, focused on research conducted in China, Croatia, Cyprus, Germany, Greece, Italy, Romania, Serbia and Turkey. Deepa et al. [19] presented the global distribution in terms of donkey milk production, and according to them, the largest producer in the world is India, followed by the USA, Pakistan, China, Brazil, Germany, Russia, France, Turkey, and Colombia.

## 3 Heavy metals

Heavy metals, including lead (Pb), cadmium (Cd), mercury (Hg), arsenic (As), nickel (Ni), vanadium (V), antimony (Sb), and barium (Ba) are environmental pollutants that pose serious risks to human health. Their presence in the environment, primarily due to anthropogenic activities such as industrial processes [28], agricultural practices [29], and waste disposal [30] has raised significant public health concerns. These metals can enter the food chain through various routes, including contaminated feed, water, and soil, thereby posing significant risks to both human health and the ecosystem. These elements in milk pose significant health risks, particularly to vulnerable populations such as infants, children, pregnant women, immunocompromised and old people. Chronic exposure to even low levels of toxic metals can lead to various health issues. Pb is a potent neurotoxin, particularly harmful to children and pregnant women [31]. It could lead to neurodevelopmental disorders, cognitive impairment, and hypertension [32]. Cd is known for its nephrotoxic and carcinogenic properties [33]. It can enter the food chain through contaminated soil and water [34]. Hg exposure, particularly in its organic form (methylmercury), can lead to severe neurological and developmental issues as well as immune dysfunction [35]. As, especially inorganic arsenic, is a known carcinogen. Furthermore, it can lead to skin lesions and cardiovascular diseases [36]. It can contaminate feed and water supplies and subsequently enter the milk of animals consuming contaminated feed or drinking contaminated water [37]. Furthermore, heavy metals like nickel (Ni), vanadium (V), antimony (Sb) and barium (Ba) can exert toxic effects as they accumulate in the body via contaminated feed, soil, and water [38,39]. Donkey milk can become contaminated with these elements, raising concerns about food safety and public health. While extensive research has focused on toxic metal contamination in cow, goat, and sheep milk [[40], [41], [42]], the occurrence of these contaminants in donkey milk has received less attention [[43], [44], [45]]. However, the contamination of donkey milk with heavy metals is a growing concern, as these metals can accumulate in the body over time, leading to above mentioned health issues. Heavy metal contamination in donkey milk is of particular concern due to the unique consumer groups that rely on it for its hypoallergenic properties and nutritional benefits. Donkey milk is often consumed by individuals with specific health conditions, such as those with cow's milk allergies, weakened immune systems, or other dietary sensitivities, making them especially vulnerable to contaminants. For these populations, donkey milk is a vital alternative that supports their health, but its safety must be ensured. Even low levels of heavy metals can pose significant health risks to sensitive groups, potentially leading to adverse effects on neurological, immune, and developmental functions, especially in children, the elderly, and individuals with compromised health. Given the importance of donkey milk in these contexts, the potential for bioaccumulation of heavy metals such as Pb, Cd, or Hg requires urgent attention. Vulnerable consumers may be more susceptible to the toxic effects of these contaminants due to their health conditions, increasing the risk of long-term damage from chronic exposure. By studying heavy metal contamination in donkey milk, we can better safeguard the health of these sensitive populations and ensure that the milk remains a safe, beneficial alternative. The above-mentioned facts highlight the need for rigorous monitoring and research, as well as the implementation of best practices to reduce contamination throughout the production process.

### 3.1 Sources, routes and pathways of contamination

As with other animals, the bioaccumulation of heavy metals in donkey milk can occur through various exposure routes, including contaminated feed, water, and environmental exposure. Previous studies have documented the bioaccumulation of heavy metals in living organisms, emphasizing the potential risks associated with contaminated sources [46]. It is important to note that donkeys are monogastric animals, unlike cows, which are ruminants. This fundamental difference influences the carry-over of toxic substances from feed to milk, as the digestive processes in monogastric animals like donkeys may result in different rates or mechanisms of heavy metal absorption and excretion. While specific studies on the bioaccumulation pathways of heavy metals in donkeys are limited, hypotheses have been proposed in the literature suggesting that contaminated feed and water are primary sources. However, the pathways may differ from those in cows due to these physiological differences. Further research is needed to better understand these processes in donkeys, but it is reasonable to hypothesize that environmental exposure and contaminated equipment may also contribute to bioaccumulation in donkey milk. Heavy metals can be present in soil, water, and air due to natural occurrences or anthropogenic activities. While specific literature on heavy metal contamination in donkey milk is limited, it is reasonable to assume that, like other grazing animals, donkeys may accumulate these elements in their bodies if they graze on contaminated pastures or drink polluted water, which can then be excreted in their milk [46]. Contaminated feed is also considered a potential source of heavy metal exposure for donkeys [47]. Donkeys consuming such feed can transfer heavy metals into their milk. However, it is important to acknowledge that these assumptions are based on the limited available research specific to donkeys, and further studies are needed to confirm these processes in donkey milk production. Comparisons with cattle are speculative and should be interpreted cautiously, as donkeys may have different physiological and metabolic responses. Contamination can also occur during milk handling and processing if equipment or containers are made from materials that leach heavy metals.

### 3.2 Regulatory and legislative framework

Numerous countries and international organizations have established maximum levels (MLs) for toxic elements in raw milk to ensure its quality and safety. The European Union (EU) and the Codex Alimentarius Commission (CAC) have established the Maximum Level (ML) for Pb at 0.02 mg/kg, as per the updated standards amended in 2023. In China, the MRLs for Pb, chromium (Cr), Hg and As are 0.05, 0.3, 0.01, and 0.10 mg/kg, respectively [[48], [49], [50]]. In the Republic of Serbia, the Maximum Residue Limit (MRL) for Pb is set at 0.02 mg/kg, while there is no MRL established for Cd, Hg and As in milk [51], which is in line with European regulations. Although specific MLs for toxic elements in donkey milk have not been specifically set, Commission Regulation (EU) [49] establishes the ML for Pb in ‘raw milk, heat-treated milk, and milk for the manufacture of milk-based products.’ According to this regulation and as defined in Regulation (EC) No. 853/2004 [52], ‘raw milk’ refers to milk from farmed animals, which includes dairy donkeys. Therefore, the MLs for Pb in bovine milk also apply to donkey milk. Current EU and Serbian legislations specify a maximum level only for Pb, set at 0.02 mg/kg (w/w). (Table 1).

**Table 1:** Maximum (residue) levels for heavy metals in milk∗ (EU, CAC, Serbia, China, Romania).

Element	Maximum (residue) level (mg/kg)	Remarks	Regulation
Lead (Pb)	0.02	The maximum level applies to the wet weight.	[48,49,51] [51]
Lead (Pb)	0.05		[50]
Chromium (Cr)	0.3		
Mercury (Hg)	0.01		
Arsenic(As)	0.1		
Cadmium (Cd)	0.05	Romania	[53]

∗Raw milk, heat-treated milk and milk for the manufacture of milk-based products.

### 3.3 Concentrations of heavy metals in donkey milk: a review of the available scientific literature

It has already been mentioned that when it comes to contamination of donkey milk with heavy metals, there is not much data available in the literature. The results of previous studies are presented in Table 2.

**Table 2:** Results of the scientific research on the presence of heavy metals in donkey milk.

Country/No of samples	Method	Element	Range (μg/kg)	Mean (μg/kg)	Reference
Italy (Sicili, Calabria, Emilia-Romagna)/45	ICP-MS∗	As	13.9–142.5	62.5 ± 31.1	[38]
		Cd	1.1–20.4	6.9 ± 4.88	
		Hg	0.05–1.90	0.88 ± 0.45	
		Ni	10–90	34 ± 20	
		Pb	2.43–59.79	13.88 ± 9.87	
		Sb	13.30–59.38	33.75 ± 12.17	
Turkey/17	ICP-MS∗	Ni	<LOD (1) <LOD (1) <LOD (1) <LOD (1)		[39]
		Cd			
		V			
		Ba			
Romania (Sălaj and Cluj)/40	ICP-MS∗	Pb	19.59–48.2	/	[53]
		Cd	4.19–6.89	/	
Italy (Reggio Emilia province) (breed:Martina-Franca)/112	ICP-MS∗	Pb	<0.42–12.5	3.2 ± 2.7	[54]
		V	<0.03–0.54	89.2 % of samples was < LOD	
		As	<0.03–0.15	80.3 % of samples was < LOD	
		Cd	<0.03–0.51	92.8 % of samples was < LOD	

∗ICP-MS - Inductively Coupled Plasma Mass Spectrometry; Pb-lead; Cd – cadmium; Ni – nickel; V – vanadium; Ba – barium; Sb – antimony; LOD – limit of detection.

Research on heavy metal levels in donkey milk is sparse, indicating a need for more comprehensive studies. Potorti et al. [38] found that most donkey milk samples did not show levels of residues harmful to human health. Specifically, Hg and As were both within acceptable limits. However, Cd and Pb occasionally exceeded safety thresholds, particularly concerning children. Pb levels in 11 % of all milk samples exceeded the European limit of 0.02 mg/kg. Specifically, 20 % of samples from one farm and 7 % from the other two farms were above this threshold. Pb contamination in milk can arise from several sources, including consumption of forages grown near roadsides, livestock exposure to polluted environments in densely populated areas, water contamination due to aging pipes or storage tanks containing Pb, and soil pollution from the use of pesticides and synthetic fertilizers, along with the influence of local climatic conditions [38]. Sb ranged from 28.77 to 38.04 μg/kg and Ni from 30.00 to 36.00 μg/kg, but direct comparisons were not possible due to insufficient parameters for comparison. According to Fantuz et al. [54], consistent with low levels found in drinking water and feed, potentially toxic elements in donkey milk were minimal, posing no health risks for human consumption. While specific correlations between milk and serum concentrations of V, As, Cd, and Pb were not defined, it was noted that Pb levels in donkey milk were higher than those in serum. Potorti et al. [38] identified significant seasonal fluctuations in the concentrations of metals in milk and feed samples; however, these variations did not consistently align between the two types of samples. This discrepancy suggests that environmental conditions influencing intake may play a role in these differences, rather than being attributed solely to the composition of forages and feed. Most examined samples did not show residues that pose a risk to human health. Specifically, levels of Hg and As fell within acceptable limits, whereas Cd and Pb occasionally exceeded the established benchmarks, particularly for children [38]. The presence of heavy metal residues may also reflect the sanitary conditions of milk and serve as an indirect indicator of environmental pollution in its production area [55].

### 3.4 The preventive measures, management practices on donkey farms and monitoring programmes

Strategies for minimizing heavy metal contamination in donkey milk should encompass a comprehensive approach that integrates proper risk assessment as a foundational element. Risk assessment is critical to identifying potential sources of contamination throughout the production chain, allowing for targeted interventions. This process is integral to the effective implementation of best practices such as Good Agricultural Practices (GAP) and Good Manufacturing Practices (GMP), both of which are recognized for their role in promoting food safety and minimizing contamination risks in agricultural and food production settings [56]. GAP emphasizes the importance of managing farm inputs and production processes to reduce the likelihood of contaminants, including heavy metals, entering the food chain. Key aspects of GAP that contribute to minimizing heavy metal contamination in donkey milk include proper management of soil quality, water sources, and feed [45]. Monitoring and controlling soil and water contamination are essential, as these are common pathways through which heavy metals can accumulate in forage consumed by donkeys [43]. Furthermore, GAP promotes the use of non-toxic fertilizers and pesticides, which can directly reduce the risk of introducing heavy metals into the animals' diet [57]. GMP focuses on ensuring that processing, handling, and storage conditions for donkey milk are designed to prevent contamination [56]. This includes maintaining hygienic facilities, using equipment made from materials that do not leach harmful substances, and conducting regular monitoring for contaminants such as heavy metals. Proper cleaning protocols for milking equipment and storage containers, as well as monitoring the milking environment, can mitigate the risk of contamination [58]. GMP also encourages ongoing employee training on contamination prevention and awareness of potential sources of heavy metals.

The Codex Alimentarius Standards, a collection of internationally recognized food safety standards, provides additional guidance on minimizing contamination risks. In particular, the Codex highlights the need for systematic control measures throughout the production chain. Specific standards, such as the “Code of Practice for the Prevention and Reduction of Lead Contamination in Foods” [59], and “Code of Practice for Source Directed Measures to Reduce Contamination of Foods with Chemicals” (CAC/RCP 49-2001) [60], suggest measures to prevent or reduce contamination in animal feed and food products. These include regular testing of feed for contaminants, ensuring safe storage conditions for feed and food products, and adhering to established maximum allowable limits for heavy metals in feed and foodstuffs. Additionally, adopting these standards into donkey farming practices can provide a robust framework for minimizing risks associated with heavy metals.

By integrating proper risk assessment with the systematic application of GAP, GMP, and relevant Codex Alimentarius standards, farmers can better mitigate the risk of heavy metal contamination in donkey milk. This approach ensures a holistic and proactive strategy that safeguards both the health of donkeys and the quality of milk produced, while also ensuring compliance with international food safety standards. This also includes using equipment and containers made from non-toxic materials, maintaining hygienic conditions, and systematically testing milk for heavy metals at critical points in the production chain: before and after milking, to ensure the donkey's health and the cleanliness of the milking environment; during milk storage, to monitor potential contamination during storage; and prior to distribution, to confirm the milk meets safety standards before reaching consumers [61]. A systematic approach to monitoring at these key stages enhances food safety and ensures the integrity of donkey milk throughout the production process. Establishing maximum levels for heavy metal contamination alone may not be sufficient to effectively manage the risks associated with heavy metal residues in donkey milk. Therefore, a more robust approach is needed, incorporating comprehensive risk management strategies. Additionally, the HACCP system should be implemented at stages beyond primary production. Specifically, the following elements are crucial: Conducting Hazard Analysis (identifying critical points in the production and processing stages where contamination may occur); Establishing Control Measures (effectively mitigating identified hazards); Monitoring and Verification (ensuring compliance with established safety standards throughout the supply chain). These steps will contribute significantly to the effective control of heavy metal hazards in donkey milk, ultimately enhancing food safety for consumers.

## 4 Aflatoxin M1 (AFM1)

Aflatoxins are a group of mycotoxins produced primarily by *Aspergillus flavus* and *Aspergillus parasiticus* [62,63]. These toxins pose significant health risks to humans and animals due to their potent carcinogenic, mutagenic, and immunosuppressive properties [64]. The presence of AFM1 in milk poses serious health risks to consumers. AFM1 is classified as a Group 2B carcinogen by the International Agency for Research on Cancer (IARC), indicating it is possibly carcinogenic to humans [65]. Additionally, AFM1 has been associated with growth impairment in children and various immunotoxic effects [66]. Given the increasing use of donkey milk in infant nutrition and as a dietary supplement for individuals with allergies to cow milk [67,68], the potential health risks posed by AFM1 contamination cannot be overlooked. While extensive research has been conducted on the presence of AFM1 in commonly consumed milk such as cow and goat milk [69,70], limited studies have specifically focused on the presence of AFM1 in donkey milk. However, the increasing popularity of donkey milk due to its hypoallergenic properties and nutritional benefits [15] necessitates a thorough understanding of potential AFM1 contamination.

### 4.1 Sources, routes and pathways of contamination

Among the various aflatoxins, aflatoxin M1 (AFM1) is a major metabolite of aflatoxin B1 (AFB1), which is excreted in the milk of lactating animals that have ingested AFB1-contaminated feed [71]. The metabolic conversion of AFB1 to AFM1 in the liver, followed by excretion into milk [72], constitutes the main pathway of contamination. Donkeys, like other mammals, can ingest AFB1 through contaminated feed, hay, or grains [73]. Once ingested, AFB1 is metabolized in the liver, where it can be converted into AFM1, which is then excreted into milk. However, the efficiency of this metabolic conversion and the carry-over of aflatoxins into milk can vary depending on species, diet, and individual metabolic rates. According to Gross et al. [73], factors such as the bioavailability of AFB1 in donkeys, their digestive physiology as monogastric animals, and their ability to metabolize and excrete toxins may influence the extent of AFM1 contamination in donkey milk. While donkeys are believed to have a lower transfer rate of aflatoxins into milk compared to ruminants, further studies are needed to fully understand these mechanisms and assess the potential risks of aflatoxin contamination in donkey milk [71].

### 4.2 Regulatory and legislative framework

Regulatory agencies worldwide have established maximum allowable limits for AFM1 in milk to safeguard public health. For example, the European Union has set a ML of 0.05 μg/kg for AFM1 in ‘raw milk, heat-treated milk, and milk for the manufacture of milk-based products,’ which is applicable to donkey milk [49]. Although the stricter limit of 0.025 μg/kg for AFM1 applies specifically to infant formulae, follow-on formulae, and young-child formulae, we need to mention these values to highlight the heightened safety standards for vulnerable populations, such as infants and young children. While these limits do not apply directly to donkey milk, their inclusion underscores the rigorous regulatory framework surrounding AFM1 levels in dairy products and emphasizes the importance of monitoring donkey milk, particularly given potential overlaps in consumer groups. The U.S. Food and Drug Administration (FDA) has set a maximum allowable limit for AFM1 in milk at 0.5 μg/kg [74]. The Food and Agriculture Organization (FAO) and the World Health Organization (WHO) recommend a maximum limit of 0.05 μg/kg for AFM1 in milk to ensure food safety. Additionally, the Codex Alimentarius, which provides international food safety standards, has established a similar limit of 0.05 μg/kg for AFM1 in milk products, including raw and heat-treated milk [75]. Furthermore, Commission Regulation (EU) No. 574/2011 specifies the limit for AFB1 at 20 μg/kg for feed materials, 10 μg/kg for complementary and complete feed, and 5 μg/kg for compound feed intended for dairy cattle and calves, dairy sheep and lambs, dairy goats and kids, piglets, and young poultry [76]. The European Union legislation establishes a maximum level of Ochratoxin A in food for special medical purposes intended for infants and young children, set at 0.5 μg/kg. This limit applies to milk, milk products, and similar products that are ready to use (either placed on the market as such or after reconstitution as instructed by the manufacturer). For products other than milk, the maximum level is based on dry matter content [49]. In discussing Ochratoxin A limits, it is important to emphasize the relationship between feed safety and the quality of donkey milk. Contaminated compound feeds intended for dairy cattle and calves can pose risks for other dairy products, including donkey milk. While donkey milk has unique characteristics, it can still be affected by the same environmental and dietary factors that influence the quality of milk from other species. By including information about the maximum levels of Ochratoxin A in these feeds, we aim to highlight the broader context of chemical contamination and the importance of monitoring feed safety to prevent the transfer of contaminants into milk. This underscores the potential risk factors that can affect the safety and quality of donkey milk.

### 4.3 Occurrence of AFM1 in donkey milk: a review of the available scientific literature

Studies on the presence of AFM1 in donkey milk are quite limited, and many have analysed only a small number of samples. These studies generally report few or no positive samples and low concentrations of the mycotoxin, as shown in Table 3.

**Table 3:** Results of the scientific research on the presence of AFM1 in donkey milk.

Country/Breed/No of samples	Period	Method	Number of Positive Samples	Average Concentration/Range (μg/kg)	Reference
Germany/Baudet du Poitou/6		EIA	0	/	[73]
Italy (Western Sicily)/84	2013–2016	ELISA	0	/	[77]
Italy/Romagnola/6		HPLC-FLD	100 % after administration of contaminated feed	0.03	[78]
Serbia/5	February–May 2013	ELISA	60 % positive (3/5)	0.02 ± 0.02/range: 0.005–0.035	[79]
Croatia/14	July–September 2013	EIA	100 %	0.005 ± 0.001/range: 0.003–0.01	[80]
Greece/36	2015	ELISA	5 out of 36 samples (13.9 %)	range 0.006–0.03	[81]
Northern Italy/Martina Franca, Amiata, Sant’ Andrea, San Domenico, Argentato di Sologno, Sardo, Ragusano, Poitou/63	June and November 2015	HPLC-FLD	62 samples < LOQ	1 sample between LOD - and LOQ (0.004)	[82]
Greece and Cyprus/90		EIA	0	/	[83]
China (Xinjiang)/70	January–March 2022	ELISA	0	/	[84]

It is also interesting to mention that in a study conducted by Capriotti et al. [85], where liquid chromatography-mass spectrometry (UHPLC-ESI-MS/MS) was used, five samples of donkey milk were tested for the presence of mycotoxin zearalenone (ZEN) and all samples were below the detection limit of 20 ng/kg. Gross et al. [73] examined the concentrations of ochratoxin and zearalenone in 6 Baudet du Poitou donkeys and also found no positive samples. Available data suggest that the levels of AFM1 in donkey milk can vary widely, influenced by factors such as geographical location, feeding practices, seasonality and weather conditions [79,80]. Studies have shown that AFM1 levels can be higher in regions with warm and humid climates that favour fungal growth [74]. Moreover, in specific climatic conditions, cereals are particularly susceptible to contamination by aflatoxin B1 and B2 [78]. Kos et al. [79] explained the occurrence of aflatoxin M1 in donkey milk by the fact that the climatic conditions in Serbia during 2012 were highly unfavourable, with very hot and dry weather leading to increased prevalence of mycotoxins in corn and animal feed in general. The type and quality of feed provided to donkeys significantly impact AFM1 levels in their milk [73,86]. In the study conducted by Tozzi et al. [78], after feeding donkeys with feed contaminated with mycotoxins, residues of aflatoxin M1 were found in milk samples. Malissiova and Manouras [81] found no statistically significant differences between seasons or types of feed used in donkey farming (P > 0.05). Donkey milk in Greece shows very low levels of aflatoxin M1, likely due to the specific feeds used for raising donkeys and the minimal carry-over of aflatoxin B1 to M1 in donkeys. Cammilleri et al. [77] did not detect the presence of aflatoxin M1 in samples of donkey milk, attributing this to the fact that the animals sampled were grazing. Donkey feed primarily consists of forage, so to boost milk production, it's essential to supplement their diet with energy sources like cereals. The fact is that donkeys are primarily grazing animals, and their main diet consists of grass and hay, which significantly contributes to reduced detection of aflatoxin M1 in their milk compared to milk from other animal species [8,82]. Gross et al. [73] also tested the feed consumed by donkeys, and it tested negative for the presence of AFB1. Furthermore, Altafini et al. [82] explain the low levels of M1 in donkey milk with the fact that the donkeys spend most of the year on pasture, and they are only exposed to aflatoxins through the feed provided in the evening when they return to the stables. The near absence of the toxin in the milk suggests that the feed is safe and consistently monitored. Organic and well-stored feeds tend to have lower levels of AFB1 contamination compared to conventional feeds that may be improperly stored or sourced from areas with high fungal prevalence. Furthermore, the very low casein content in donkey milk, compared to cow milk [4] is another possible explanation for low levels of AFM1. This toxin has a high affinity for casein [87], so in milk from animals exposed to aflatoxins through feed, there might be a correlation between AFM1 levels and casein percentage. Another possible explanation is the minimal transfer of AFM1 into donkey milk. While there is extensive literature on lactating cows, there is limited information on the transfer of AFM1 into donkey milk. The only study available is by Tozzi et al. [78] which examined the levels of AFM1 in donkey milk after feeding donkeys with corn naturally contaminated with AFB1 and AFB2. Over 12 days, six donkeys were given a diet containing 202 μg of AFB1 and 11 μg of AFB2. Administration of corn naturally contaminated with AFB1 and AFB2 resulted in increased AFM1 content in the milk of all animals, with carry-over rates of 0.02 % for AFM1. Notably, this carry-over percentage is relatively low compared to other species, particularly ruminants [88]. Research indicates that AFM1 carry-over is generally lower in monogastric animals, such as donkeys, compared to ruminants [78]. This distinction highlights the differences in AFM1 bioaccumulation across species, suggesting that donkeys may be less susceptible to higher levels of AFM1 contamination.

### 4.4 The preventive measures, management practices on donkey farms and monitoring programmes

To minimize AFM1 contamination in donkey milk, several strategies can be implemented: GAP, GMP and Regulatory Monitoring [86]. Adopting GAP can significantly reduce the risk of AFB1 contamination in animal feed [86,89]. These practices include proper drying and storage of feed, regular monitoring for fungal contamination, and the use of antifungal agents when necessary. GMP in the dairy industry involves rigorous quality control measures during milk production, processing, and storage. Implementing these practices helps prevent AFM1 contamination and ensures the safety of the final product [90]. Regular monitoring and surveillance of AFM1 levels in donkey milk by regulatory authorities can help identify contamination sources and enforce compliance with safety standards. Establishing national and international regulations specific to donkey milk can further enhance consumer protection. Implementing good agricultural and storage practices can thus mitigate the risk of AFM1 contamination in donkey milk. While donkey milk is regarded as safe concerning aflatoxin M1, ongoing monitoring is recommended since aflatoxin levels in feed of plants origin can be influenced by various factors.

## 5 Organochlorine pesticides (OCPs) and polychlorinated biphenyls (PCBs) levels

Organochlorine pesticides (OCPs) and PCBs are persistent organic pollutants (POPs) known for their environmental stability, lipophilicity, and potential to bioaccumulate in the food chain. These compounds have been widely used in agriculture and industry due to their effectiveness and durability [91]. However, their persistence in the environment and toxicological effects on human health has led to global concerns and regulatory actions to limit their use and presence in food products. Donkey milk, valued for its nutritional and hypoallergenic properties, can be affected by organochlorine compounds environmental pollution [92]. The presence of OCPs and PCBs in donkey milk poses significantly lower health risks in comparison with cow milk, particularly to vulnerable populations such as infants, children, and pregnant women [8]. Notwithstanding, chronic exposure to these compounds can lead to various adverse health effects. OCPs can lead to carcinogenicity, endocrine disruption, neurotoxicity, and reproductive toxicity [93]. Long-term exposure to OCPs like DDT has been linked to increased risk of cancers, endocrine disorders, and neurodevelopmental issues [94]. PCBs can lead to carcinogenicity, immunotoxicity, mutagenicity, neurotoxicity, and endocrine disruption [95]. Furthermore, PCBs are associated with developmental and behavioural problems in children, immune system impairment, and increased risk of cancer [96]. Given the rising popularity of donkey milk for its health benefits, ensuring its safety by minimizing OCP and PCB contamination is crucial.

### 5.1 Sources, routes and pathways of contamination

Organochlorine pesticides (OCPs), such as DDT, lindane, and aldrin, have historically been widely used in agriculture for pest control. Despite being banned or restricted in many countries, their residues persist in the environment due to their long half-lives [97]. OCPs can contaminate donkey milk through several pathways: environmental exposure, contaminated feed, and bioaccumulation. Residues from OCPs can enter the soil, water, and air, where they may be ingested by donkeys while grazing or drinking contaminated water. Contaminated feed, which can arise from direct pesticide application or environmental deposition, is a primary source of OCPs in donkey milk [98]. Due to their lipophilic nature, OCPs tend to accumulate in animal fat and are transferred to milk during lactation.

Similarly, polychlorinated biphenyls (PCBs) pose another significant contamination risk. These chemicals were used in various industrial applications, including electrical equipment, heat transfer fluids, and plasticizers [99]. Although their production has been banned in many countries due to environmental persistence and associated health risks [100], PCBs continue to contaminate donkey milk through atmospheric deposition and contaminated feedstuffs [101]. Historical use and improper disposal of industrial wastes contribute to PCB residues found in soil, water, and air [99]. Donkeys that consume contaminated feed can bioaccumulate PCBs, which are subsequently transferred to their milk. Additionally, PCBs can travel long distances in the atmosphere, depositing on pastures and water bodies, further contributing to the contamination of donkey milk.

### 5.2 Regulatory and legislative framework

Maximum residue levels (MRLs) for pesticide residues in plant and animal products are established to protect consumers and facilitate trade. We acknowledge that there are currently no maximum residue levels (MRLs) for pesticides specifically pertaining to donkey milk in either national or EU legislation. In the European Union, legislation sets the MRLs for the pesticides dichloro-diphenyl-trichloroethane (DDT) and endrin in milk at 40 ng/g and 0.8 ng/g, respectively [102]. EU legislation harmonises and simplifies pesticide MRLs, and sets a common EU assessment scheme for all agricultural products for food or animal feed [49]. The European Union runs a coordinated multiannual control programme that mandates Member States to annually sample, analyse, and test a variety of dietary products for specific pesticides. This is done to ensure compliance with MRLs and to evaluate consumer exposure to these chemicals. The European Food Safety Authority (EFSA) gathers and publishes the results each year [103]. Additionally, Member States operate their own risk-based national programmes alongside the EU's coordinated efforts. The maximum amount of organochlorine pesticides in milk in Serbia is regulated by the Regulation on Maximum Residue Levels of Plant Protection Products in Food and Feed [104], while the maximum allowable (acceptable, permitted) concentrations of PCBs are prescribed by the Regulation on Maximum Concentrations of Certain Contaminants in Food [51]. The maximum level of PCB is regulated in EU by the Commission Regulation 915/2023. The values of Maximum permitted concentrations of PCBs prescribed in the EU and Serbia are shown in Table 4.

**Table 4:** The maximum levels of PCBs in milk in the EU and Serbia.

Maximum level			Regulation
Sum of dioxins (pg WHO-PCDD/F-TEQ/g)	Sum of dioxins and dioxin-like PCBs (pg WHO-PCDD/F-PCB-TEQ/g)	Sum of non dioxin-like PCBs is of PCB28, PCB52, PCB101, PCB138, PCB153 and PCB180 (ICES - 6).	
2.5 pg/g fat	5.5 pg/g fat	40 ng/g fat	[51]
2.0 pg/g fat	4.0 pg/g fat	40 ng/g fat	[49]

### 5.3 Concentrations of OCPs and PCBs in donkey milk: a review of the available scientific literature

Studies on the occurrence of OCPs and PCBs in donkey milk are very limited and available data are shown in Table 5. According to our knowledge, research related to the presence of OCPs and PCBs in donkey milk has been published in only two papers [92,105].

**Table 5:** Results of the scientific research on the presence of OCP and PCBs in donkey milk.

Country	Method	Residue	% - percentage of positive samples.	Range (ng/g fat)∗	Reference
Italy (Sicily, Calabria and Emilia-Romagna)	GC-ECD and GC-MS	OCPs			[105]
		Aldrin	13.33	n.d.∗∗-2.51 ± 0.31	
		Dieldrin	6.66	n.d.- 14.31 ± 0.72	
		4,4′-DDE	57.67	1.54 ± 0.14–45.69 ± 1.36	
		ΣPOC	57.67	1.54–60	
		PCB			
		PCB 28 (T)	15.33	1.98 ± 0.03–60.00 ± 1.55	
		PCB 52 (T)	20	5.62 ± 0.68–94.43 ± 1.93	
		PCB 101 (T)	29	n.d.- 28.86 ± 1.21	
		PCB 81 (DL)	29	n.d.- 6.64 ± 0.74	
		PCB 77 (DL)	26.67	n.d. - 20.41 ± 1.11	
		PCB 123 (DL)	6.67	n.d. - 4.32 ± 0.23	
		PCB 118 (T/DL)	42.33	0.88 ± 0.04–3.14 ± 0.04	
		PCB 114 (DL)	13.33	n.d.- 2.88 ± 0.17	
		PCB 153 (T)	64.33	1.28 ± 0.23–11.03 ± 0.51	
		PCB 138 (T)	60	1.34 ± 0.17–4.33 ± 0.32	
		PCB 157 (DL)	4.33	n.d.- 0.99 ± 0.06	
		PCB 180 (T)	64.33	0.66 ± 0.06–2.88 ± 0.11	
		ΣPCB	73.33	4.91–202.6	
		Σtarget∗∗∗	73,33	3.92–178.14	
		WHO-TEQs∗∗∗∗	62.33	0.09–2.52	
Southern Italy	GC-MS	Σ6PCB	100	1.26–708.80	[92]
		Σ12PCB	97	n.d.∗∗-163.20	
		Σndl-PCB	100	1.26–872.00	
		Σdl-PCB	63.5	n.d.∗∗-211.20	
		HCB	92.75	n.d.∗∗- 48.80	
		ΣDDTs	98.25	n.d.∗∗-428.80	

∗Minimum and maximum values are expressed as mean values ± 95 % confidence interval; ∗∗n.d. = <LOQ; ∗∗∗Sum of congeners n. 28, 52, 101, 118, 138, 153, 180; ∗∗∗∗Sum of dioxin-like congeners expressed in toxic equivalents of the World Health Organization using toxic equivalency factor (WHO-TEFs).

Di Bella et al. [105] collected forty-five donkey milk samples from three conventional Italian donkey farms in the months of September and November 2011, and in January, March and May 2012. The results obtained by Di Bella et al. [105] indicated minimal differences between samples from various farms, with contamination levels remaining very low and typically below the legislative maximum residual limits of 0.04 mg/kg for DDTs, 0.006 mg/kg for aldrin with dieldrin and for PCBs. Additionally, when compared to the current Tolerable Daily Intake (TDI) values proposed by FAO, WHO and The Scientific Committee on Food (SFC) [[106], [107], [108]], the daily intake levels of POCs and PCBs in donkey milk were found to be very low for many of the examined samples. They concluded that although their findings should not be viewed as entirely alarming, the survey indicated that children consuming donkey milk are inevitably exposed to these substances. Therefore, it is essential to monitor donkey milk as functional food to understand how environmental pollution sources might elevate contamination levels. Monnolo et al. [92] emphasized that donkeys can be exposed to environmental pollutants, specifically organochlorine compounds, due to grazing. They examined 55 samples of milk from donkeys of the Ragusana, Amiatina, Martina Franca, and Sicilian Grey breeds from small conventional farms during the summer months (June–August 2016). Both mentioned studies obtained qualitatively similar results, with the presence of indicator PCBs and dichlorodiphenyldichloroethylene (p,p’-DDE) being characteristic. The concentrations of the examined pesticides were generally low in both studies, which they attributed to the limited use of pesticides in the areas they investigated. Higher concentrations of both OCPs and PCBs were measured in donkeys that were exclusively grazing compared to those that received mixed feed [92]. They emphasized the importance of their findings and the potential health risks associated with dl-PCB intake, especially for primary consumers of donkey milk like infants and children who are intolerant to cow milk and other foods, as well as elderly people. This concern is heightened by the expected rise in donkey milk consumption in Europe due to the growing prevalence of allergies to cow's milk.

### 5.4 The preventive measures, management practices on donkey farms and monitoring programmes

To minimize OCP and PCB contamination in donkey milk, several strategies can be implemented such as GAP, GMP and Regulatory Monitoring. Adopting GAP can reduce the risk of contamination from pesticides and environmental pollutants. These practices include using safe and approved pesticides, regular soil and water testing, and minimizing the use of contaminated feed. Implementing GMP in milk production and processing can prevent contamination. This includes maintaining clean and hygienic conditions, using non-toxic materials for equipment and containers, and regularly testing milk for contaminants. Establishing maximum allowable limits for these compounds in donkey milk, enforcing these standards, and regular monitoring and surveillance by regulatory authorities can help identify and control sources of OCP and PCB contamination. Measures such as GAP and GMP must be integrated with a robust risk evaluation and characterization process, as required by Regulation (EC) [109], to effectively minimize the risk of contamination from pesticides and environmental pollutants.

## 6 Residue of antibiotics and antiparasitics

The use of antibiotics and antiparasitics in livestock is a common practice to ensure animal health and productivity. However, the presence of residues from these veterinary drugs in milk poses significant public health concerns. The presence of antibiotic and antiparasitic residues in milk poses significant health risks, particularly to infants, children, and immunocompromised individuals. Potential health impacts of antibiotic residue include allergic reactions and antimicrobial resistance. Furthermore, antibiotic residues can affect the balance of gut microbiota, leading to gastrointestinal issues and other health problems. When it comes to antiparasitic residues, some can be toxic, causing acute or chronic health effects, including neurotoxicity and hepatotoxicity. Similar to antibiotics, some individuals may have allergic reactions to antiparasitic residues.

### 6.1 Sources, routes and pathways of contamination

Antibiotic residues can appear in donkey milk through treatment of bacterial infections in lactating donkeys, preventive use of antibiotics, especially when used indiscriminately or without proper veterinary guidance or through feed and water contaminated with veterinary drugs [8,110]. The Food and Drug Administration (FDA) classifies donkeys as a minor species, resulting in no drugs being specifically labelled for their use [111,112]. Medications administered to donkeys are seldom dosed based on specific evidence and are often extrapolated from dosages used for horses. This practice can pose risks, potentially compromising the effectiveness of the treatment and increasing the likelihood of side effects [113]. Consequently, medications, including antibacterial antimicrobials, are administered to donkeys in an extra-label fashion. These differences in drug metabolism highlight the clear necessity for pharmacological studies in donkey species to develop evidence-based medical treatments and establish appropriate withdrawal times [114]. There is very little data on the use of antibiotics in donkeys, and considering this, as well as their specific differences compared to horses, special attention must be paid to the withdrawal period when it comes to donkey milk [112,113]. The available literature lacks information on the presence of antibiotics in donkey milk, making it essential to conduct future research to assess the safety of donkey milk concerning antibiotic residues. This is particularly important given the fact that veterinary drugs used in donkeys are intended for horses, and future studies need to consider all the specificities of donkeys to ensure the safer and more appropriate use of medications in these animals [[115], [116], [117]].

Antiparasitic drugs, including anthelmintics and ectoparasiticides, are used to control internal and external parasites in donkeys. Data on the use of antiparasitics in donkeys is very scarce. Residues can appear in donkey milk through direct treatment of parasitic infections in lactating donkeys which can lead to residues if withdrawal periods are not followed. Moreover, residues can result from the use of antiparasitics in the environment where donkeys graze, leading to contamination through ingestion or dermal absorption [118]. Also, antiparasitic drugs used in feed or water can lead to residues in milk [119]. Nematode infections are a common health problem in donkeys [120,121]. Although ivermectin is not officially approved for use in donkeys, many donkey keepers and veterinarians currently use it to combat nematodes and arthropods, reporting excellent therapeutic outcomes [122]. Ivermectin is administered to control both internal and external parasites in various livestock and companion animal species. Its pharmacokinetic properties largely depend on the animal species, formulation, and route of administration. Due to its high lipophilicity, ivermectin accumulates in the milk of food-producing animals; therefore, its use must be avoided during the lactation period [123].

### 6.2 Regulatory and legislative framework

To ensure the safety of milk, regulatory agencies have established maximum residue limits (MRLs) for antibiotics and antiparasitics. These limits vary by country and region but generally aim to protect public health by minimizing exposure to harmful residues. The EU has stringent MRLs for antibiotics and antiparasitics in milk, enforced through regular monitoring and compliance checks [124]. The Food and Drug Administration (FDA) sets MRLs for veterinary drug residues in milk and conducts periodic testing to ensure compliance [125]. Codex Alimentarius, an international food standards body provides guidelines and MRLs for veterinary drug residues, facilitating international trade and protecting consumer health [126].

Extensive pharmacokinetic studies of ivermectin have been conducted in horses, pigs, cattle, sheep, and goats, but there is a lack of information for donkeys. As a result, the absence of a designated milk withdrawal time and maximum residue limits (MRLs) for ivermectin in donkey milk means it should not be used in lactating animals [122]. The lack of established MRLs for ivermectin in milk indicates that its use in lactating animals is not advisable and any residues in milk intended for human consumption are not permitted. Since no MRL has been set for ivermectin in donkey milk and its use in lactating donkeys is planned, any detected residues would be deemed illegal. However, in emergencies where alternative drugs are unavailable or there is a life-threatening situation for the animal, the use of ivermectin might be considered [122]. The situation is the same when it comes to antimicrobial veterinary drugs and other antiparasitics. Such use would require that the treated milk not be consumed by humans and that the withdrawal period be established by a veterinarian [122,127,128]. Ultimately, the absence of suitable veterinary drugs for species like donkeys often leads to the misuse or even illegal use of veterinary drugs. According to Regulation 470/2009/EC and Regulation (EC) No. 37/2010 that was amended on April 08, 2024 [124,129] withdrawal periods from anthelmintic drugs in dairy donkeys need to be carefully managed.

### 6.3 Concentrations of antimicrobial and antiparasitic veterinary drugs residue in donkey milk: a review of the available scientific literature

The occurrence of antibiotic and antiparasitic residues in donkey milk is not well-documented, but residues have been reported in milk from other species [130]. In a study of 31 donkey herds in Central Italy, Ragona et al. [131] found a high prevalence of strongyle parasites. Proper management of parasitic diseases is crucial, with selective control programs being preferable to minimize the use of anthelmintic drugs. For dairy donkeys, it is essential to carefully manage withdrawal periods from these drugs, adhering to EU and national regulations. Since there is no specific legislation for the production and sale of donkey milk for human consumption, biological, chemical, and physical evaluations are necessary to ensure nutritional quality and food safety. The high prevalence of strongyles in the research conducted by Ragona et al. [131] may be linked to management practices, such as free outdoor paddocks, and the absence of effective parasite control programs. Establishing a comprehensive parasite control program for food-producing animals is vital to ensure their welfare and health while reducing the reliance on anthelmintic drugs.

### 6.4 The preventive measures, management practices on donkey farms and monitoring programmes

To minimize antimicrobial and antiparasitic veterinary drug residues in donkey milk, several strategies can be implemented, including Good Veterinary Practices (GVP), Good Agricultural Practices (GAP), Good Manufacturing Practices (GMP), and regulatory monitoring. Adopting GVP, such as proper diagnosis, treatment adherence to withdrawal periods, and responsible drug use, can reduce contamination risks. GAP focuses on ensuring clean feed and water, while regular testing can prevent disease and contamination. GMP involves rigorous quality control in milk production and processing, including regular residue testing and maintaining hygienic conditions. Establishing and enforcing maximum residue levels (MRLs) and conducting regular regulatory monitoring can help identify and control contamination sources.

## 7 Analytical methods for detection and quantification

Accurate detection and quantification of chemical residues in donkey milk are essential for ensuring food safety and regulatory compliance. Various analytical methods are employed for this purpose such as Stripping Voltammetry, Atomic Absorption Spectroscopy (AAS), Atomic Fluorescence Spectrometry (AFS), Electrothermal Atomic Absorption Spectroscopy (ETAAS), Flame Atomic Absorption Spectrometry (FAAS), Graphite Furnace Atomic Absorption Spectrometry (GFAAS), Hydride Generation Atomic Absorption Spectrometry (HGAAS), Inductively Coupled Plasma Mass Spectrometry (ICP-MS), Inductively Coupled Plasma Atomic Emission Spectrometry (ICP-AES), Inductively Coupled Plasma Optical Emission Spectroscopy (ICP-OES), Inductively Coupled Plasma-Sector Field Mass Spectrometry (ICP-SFMS), capillary zone electrophoresis and X-ray Fluorescence (XRF) for heavy metals [38,132]; Enzyme-Linked Immunosorbent Assay (ELISA) [133], High-Performance Liquid Chromatography (HPLC) coupled with fluorescence detection (HPLC-FLD) or mass spectrometry (HPLC-MS) [134], Thin-Layer Chromatography (TLC) [135] and Liquid Chromatography-Tandem Mass Spectrometry (LC-MS/MS) [136] for AFM1; Gas Chromatography (GC) [137], HPLC [138], Solid-Phase Extraction (SPE) and QuEChERS Method [139] for OCPs and PCBs, and ELISA, HPLC, GC and LC-MS/MS for analyzing veterinary drugs in milk [140].

## 8 Conclusion

The presence of chemical hazards such as heavy metals, AFM1, OCP, PCBs, and residue of antimicrobial and antiparasitics veterinary drugs in donkey milk represents a potential food safety hazard, necessitating comprehensive monitoring and control measures. Although research on contamination in donkey milk is limited compared to other types of milk, the increasing demand for donkey milk underscores the importance of addressing this issue. Continued efforts in improving detection methods, implementing best practices in agriculture and dairy production, continued research and enforcing regulatory standards will be crucial in safeguarding public health and ensuring the safety of donkey milk as a nutritious and hypoallergenic food source. Future research should focus on expanding the existing knowledge regarding chemical contamination in donkey milk, including more extensive studies, risk assessment studies, and the development of innovative preventive measures. By addressing these gaps, we can better protect consumers and promote the safe consumption of donkey milk.

## CRediT authorship contribution statement

**Dragana Ljubojević Pelić:** Writing – review & editing, Writing – original draft, Visualization, Methodology, Investigation, Formal analysis, Data curation, Conceptualization. **Sava Lazić:** Resources, Funding acquisition. **Milica Živkov Baloš:** Writing – review & editing, Supervision, Resources, Project administration, Funding acquisition.

## Data availability

No data was used for the research described in the article.

## Declaration of competing interest

None.

All authors disclose any financial and personal relationships with other people or organizations that could inappropriately influence (bias) their work.
